# Wound dressings: curbing inflammation in chronic wound healing

**DOI:** 10.1042/ETLS20200346

**Published:** 2021-07-01

**Authors:** Davide Vincenzo Verdolino, Helen A. Thomason, Andrea Fotticchia, Sarah Cartmell

**Affiliations:** 1 Department of Materials, School of Natural Sciences, Faculty of Science and Engineering and The Henry Royce Institute, Royce Hub Building, The University of Manchester, Manchester, U.K.; 2 3M, Medical Solutions Division, Knutsford, U.K.; 3 School of Health Sciences, Faculty of Biology, Medicine and Health, The University of Manchester, Manchester, U.K.

**Keywords:** chronic ulcers, collagen, inflammation, wound dressing, wound healing

## Abstract

Chronic wounds represent an economic burden to healthcare systems worldwide and a societal burden to patients, deeply impacting their quality of life. The incidence of recalcitrant wounds has been steadily increasing since the population more susceptible, the elderly and diabetic, are rapidly growing. Chronic wounds are characterised by a delayed wound healing process that takes longer to heal under standard of care than acute (i.e. healthy) wounds. Two of the most common problems associated with chronic wounds are inflammation and infection, with the latter usually exacerbating the former. With this in mind, researchers and wound care companies have developed and marketed a wide variety of wound dressings presenting different compositions but all aimed at promoting healing. This makes it harder for physicians to choose the correct therapy, especially given a lack of public quantitative data to support the manufacturers’ claims. This review aims at giving a brief introduction to the clinical need for chronic wound dressings, focusing on inflammation and evaluating how bio-derived and synthetic dressings may control excess inflammation and promote healing.

## Introduction

Wound healing is a complex process that involves numerous cell types, cytokines, chemokines, growth factors and extracellular matrix (ECM) components, which work synergistically to achieve healing [1,2]. It consists of four overlapping stages: haemostasis, inflammation, proliferation and remodelling, each with its own function and role valuable for the next phase to occur smoothly and with no delay [1,2]. In a healthy wound (i.e. acute wound), these stages usually occur with no obstacle, resulting in a completely healed wound with minimal scar tissue, albeit with slightly reduced mechanical properties (∼80%), when compared with the skin before injury [3].

A chronic wound is defined as a wound that has failed to proceed through the wound healing process readily, and it does not heal within 3 months under standard of care [2,4]. It usually results when patients present comorbidities such as diabetes, obesity, immune system deficiencies, peripheral vascular disease and cardiopulmonary disease [1,2,5]. These wounds often stall in the inflammatory stage, whereby excess and persistent inflammation creates a hostile wound environment. A wound that is slow or fails to heal is at greater risk of infection. If infection does occur, the heightened inflammation is further exacerbated [1,2]. Chronic wounds include diabetic foot ulcers (DFUs), pressure ulcers (PUs) and leg ulcers (LUs), which include venous leg ulcers, arterial leg ulcers and ulcers of mixed aetiology [4].

Wound care and chronic wounds, in particular, represent a health economic burden worldwide, accounting for an NHS annual expenditure in the management of chronic wounds of £5.3billion with a mean cost of £3700 per unhealed wound [6,7]. With such a demand, the global advanced wound dressings market targeting chronic and surgical wounds is expected to exceed ∼£16.5 billion by 2024 [5]. Furthermore, as population and life expectancy increases, the effect of wound management and chronic wounds on global health services will increase further, calling for the development of therapies that can relieve both patients and healthcare systems of this economic and societal burden [8,9].

Two main factors that are usually addressed as common underlying issues in non-healing wounds are inflammation and infection [2]. Infection occurs when immune cells fail to readily eliminate harmful microorganisms infiltrating the wound [10]. Different grades of infection require different therapies and present different degrees of severity: local infection (when infection is contained only at the wound site, usually easy to address); spreading infection (signs and symptoms of infection outside wound border in neighbouring tissues); systemic infection (affects the whole body and may present a severe issue for the health of the patient) [2]. Infection is a major contributing factor to the failure of a wound to heal [2].

**Figure 1.**
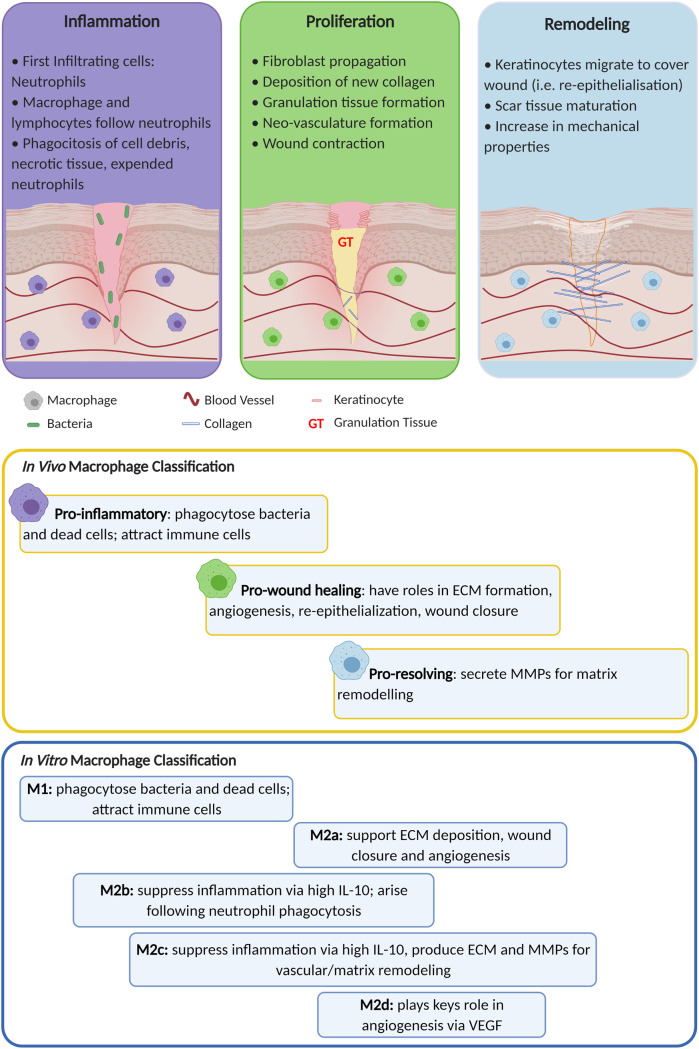
Schematic of the wound healing stages focusing on macrophage phenotypes classification *in vivo* and *in vitro* with their respective functions. Adapted with permission from [13]. Created with BioRender.com.

Inflammation represents the body's immune system response to foreign agents such as microorganisms or damaged host tissue; therefore, it is necessary and essential to achieving healing [11]. Inflammation occurs in two stages: early and late inflammation [1,2]. During early inflammation, the innate immune system is activated, and neutrophils are recruited in the wound to remove microorganisms, cellular debris and non-functional tissue [1,9,11]. Neutrophils are followed by infiltrating monocytes that differentiate into ‘M1’ pro-inflammatory macrophages as a response to pathogen-associated molecular patterns (PAMPs), danger-associated molecular patterns (DAMPs), IL-2, IFN-γ and TNF-α [9,12,13]. ‘M1’ macrophages present a high phagocytic capability, and their primary role is to remove any harmful agents. They also secrete pro-inflammatory cytokines such as IL-1, TNF-α, IL-6, IL-12, reactive oxygen species (ROS) and matrix metalloproteinase (MMPs) [9,12,13]. ROS are produced as a mechanism of killing microorganisms, but in excess, when inflammation is out of control, they cause direct tissue damage to the ECM and result in premature cell senescence [14]. MMPs degrade the damaged ECM to allow infiltration of pro-healing cells and factors [9,13]. It is thought that elevated levels of MMPs participate in stalling and delaying the wound healing process in chronic wounds [9,13]. This is because there is a delicate balance between MMPs and their inhibitors, tissue inhibitors of metalloproteinases (TIMPS) [9,13]. When this balance is tipped in favour of MMPs, degradation of healthy tissue and subsequent persistent inflammation creates a negative feedback loop that contributes to delayed healing [9,13]. In an acute wound, once inflammation is resolved and the wound is cleared of contamination, healing progresses into the proliferative stage, where granulation tissue is formed. Keratinocytes start to proliferate and migrate across the wound bed to re-epithelialise the wound [9,13]. More macrophages switch from the pro-inflammatory ‘M1’ phenotype to the pro-healing ‘M2’ phenotype, becoming the most common leukocyte in the wound [9,12,13]. At this point, a chronic wound tends to stall since ‘M1’ macrophages persist without switching to the ‘M2’ phenotype, resulting in elevated levels of pro-inflammatory cytokines produced by ‘M1’ macrophages and by the delayed removal of expended neutrophils [9,11–13]. It is worth noting that macrophages do not present a binary classification of phenotype, but rather a spectrum, as shown in Figure 1; however, in this review, ‘M1’ and ‘M2’ terminology will be used for simplicity [11,13]. A prolonged and heightened state of inflammation, no matter the cause, results in delayed healing. Various means have been proved to curb inflammation in wound healing [15,16]; in this paper, we review how wound dressings reduce excess inflammation allowing chronic wounds to heal more readily.

## Wound dressings: an introduction

The increasing prevalence of chronic wounds highlights the importance of developing innovative products to promote healing [2,17]. In a clinical setting, when a patient presents a wound, clinicians apply a set of principles that aids them to understand the wound aetiology and subsequent wound bed preparation [17,18]. These principles are represented by the acronym TIME, which is defined as Tissue assessment and management, Infection/Inflammation management, Moisture imbalance/management and Edge of wound observation and management [17,18]. Even when TIME management is followed, some wounds fail to heal and require more advanced interventions to restart the healing process [17,18].

Over time, wound management has evolved drastically, from assuming that a dry wound environment would aid healing to prioritise moisture retention when developing new wound dressings [19]. This shift in knowledge was mirrored in the shift in the type of wound dressings used. The more traditional wound dressings (e.g. gauze, lint and cotton wool), which aimed at only covering the wound, have now been replaced by sophisticated materials (e.g. hydrogels, hydrocolloids, foams, films, etc.) that aim to [19–21]:

- Provide or maintain a moist environment;
- Enhance cellular migration;
- Promote angiogenesis and new tissue synthesis;
- Allow gas exchange from/to wound;
- Maintain appropriate tissue temperature;
- Protect against bacterial infection;
- Be easily removed after healing or between dressing changes;
- Promote autolytic debridement;
- Be sterile, non-toxic and hypoallergenic.

One of the more recent advantages introduced into wound dressing formulations, and an indirect way to reduce inflammation by resolving bioburden, is the addition of antimicrobial agents such as antiseptics, antibiotics and natural products [22]. Several reviews provide additional information for a more detailed analysis of the different types of antimicrobial agents and dressings [23–25].

There are thousands of wound dressings on today's market, some claiming the same benefits but presenting different compositions [2,21]. This large number of options makes choosing an appropriate dressing an ever so complicated task for physicians [2,21,26]. Choosing an appropriate dressing is essential to achieve faster healing and depends on many factors, including the type and location of the wound, the level of exudate, the integrity of the surrounding skin, and whether the wound is infected or stalled in the inflammatory phase of healing [21,23,24,27]. A summary of different types of wound dressings is given in Table 1.

**Table 1.** Summary of different types of dressing with the corresponding description

Type of dressings	Description	Ref
Gauze	Drying Cheap May produce painful removal	[19–21]
Foams	Soft and conformable High porosity, moderately absorbent Thermal insulating Wounds may dry out if little or no exudate	[23,24]
Films	Occlusive, retains moisture No absorbent properties Protects against infection Can cause fluid collection	[19,20,102,103]
Hydrogels	Maintain moist environment Aid in autolytic debridement Rehydrates dry wound Easy removal Not suitable for heavily exuding wounds May require secondary dressing	[20,103–105]
Hydrocolloids	Occlusive Highly absorbent May cause peri-wound maceration May adhere to the wound and damage fragile tissue	[23,24,103,106]
Alginates	Moderate-highly absorbent Haemostatic Maintain moist environment Not suitable for dry wounds May require secondary dressing	[20,103,107,108]
Gelling Fibres	Moderate-highly absorbent Maintain moist environment Forms gel when in contact with exudate Easy to remove Not suitable for dry wounds	[109,110]
Superabsorbent dressings	Highly absorbent Conformable Prevents maceration Not suitable for dry wounds	[111–114]

As described before, pathologically extensive inflammation plays an essential role in the delayed wound healing process of chronic ulcers [28]. This is usually caused by multifactorial stimuli that create a hostile microenvironment (e.g. excess levels of inflammatory cells) in which the balance between pro-inflammatory mediators (e.g. chemokines, cytokines, proteases) and their inhibitors is disrupted [28]. The large number of biological entities involved in the inflammatory process makes anti-inflammatory wound dressings varied in their mechanisms of action. For example, some only address locking in exudate away from the wound bed along with the inflammatory components present in wound exudate [29,30]. Others instead focus on regulating pro- and anti-inflammatory cytokines either directly or via macrophage phenotype regulation [31–35]. Nonetheless, the ultimate aim of anti-inflammatory wound dressings is to remove the perpetuating cause and provide a healthy wound microenvironment to promote granulation tissue formation and to promote the healing processes [28].

### Bio-derived wound dressings to regulate inflammation

In the last decade, more researchers investigated bio-derived materials as building blocks for new wound dressings [36,37]. These aim to mimic the skin's ECM to provide the chronic wound with a substrate that can function as a healthy ECM or as a sacrificial scaffold for proteases degradation, thereby protecting the native ECM and rebalancing the wound microenvironment allowing for faster healing. The ECM is more than a passive physical substrate for cells; it actively participates in cell–cell communications via cell–matrix interactions, as a result of and resulting in the activation of biochemical mediators, cytokines and growth factors, which play a significant role in wound physiology [38–40]. Although bio-derived materials can provide a high degree of biomimicry, they can be limited by their reproducibility during manufacturing, given by variance between batches [37,41]. The utilisation of animal sources raises concern also from the transmission of pathogens standpoint [42].

The skin ECM comprises the epidermal ECM (i.e. a basement membrane, which separates the epidermis from the dermis) and the dermal ECM. The latter is composed of fibroblasts embedded in connective tissue fibres, interstitial fluid, cell adhesion proteins (e.g. laminin, vitronectin, fibronectin), glycosaminoglycans (GAGs) and proteoglycans [43]. Collagen is the major component of dermal ECM, accounting for ∼70% of skin (dry weight) [44]. It presents 28 genetically different variants, with type I and III variants being the most abundant in the skin [44–46].

Refer to Table 2 for a summary of currently commercially available dressings or in the research stage of development.

**Table 2.** Non-exhaustive summary of wound dressings with anti-inflammatory effects currently on the market (M) or in the research (R) stage, showing their composition and reported anti-inflammatory ability

Dressing	Composition	Anti-inflammatory effects	Market (jurisdiction) or research	Ref.
Bio-derived Dressings				
Endoform® Antimicrobial Dermal Template	Decellularised ovine forestomach matrix with 0.3%w/w silver chloride	Broad-spectrum of MMPs inactivation capability Retention of structural molecules and growth factors	M(US)	[32,57,61]
Puracol® Ultra Matrix	Decellularised porcine mesothelium matric	Retention of growth factors (FGF-basic, VEGF, and TGF-β1) MMPs inactivation capability	M(US)	[58]
Integra® Dermal Regeneration Template	Cross-linked bovine tendon collagen type I, shark chondroitin-6-sulfate GAG, silicon membrane	Chondroitin-6-sulfate GAG has anti-inflammatory properties Allows quick permeation of cells	M(US & EU)	[49–53]
OASIS® Ultra Tri-Layer Matrix	Decellularised porcine small intestinal submucosa	Retention of structural molecules and growth factors	M(US)	[54,115–117]
Apligraf®	Bovine type I collagen seeded with human neonatal fibroblasts and keratinocytes	↑ VEGF, IL-6, IL-8 ↓ fibrotic TGF-β1 Restore fibroblasts function	M(US)	[118–123]
Q-peptide	Chitosan-collagen hydrogel functionalised with QHREDGS peptide	Provide resistance to oxidative stress Induce shift in macrophage polarisation	R	[33,86–88]
Co-modified CBD-VEGF-SDF-1α collagen scaffold	Collagen scaffold modified with CBD-VEGF-SDF-1α	↓ Infiltration of ‘M1’ macrophages ↓ IL-1β and TNF-α	R	[34]
NAg-CSS	Chitosan-collagen loaded with silver nanoparticles	Modulate macrophage polarisation ↓ IL-6, TNF-α and TGF-β ↑ IL-10 and IFN-γ	R	[35]
Promogran™ Matrix	Freeze-dried matrix composed of 55% bovine type I collagen with 45% ORC	Bind and inactivate proteases by means of ORC Bind and protect naturally occurring growth factors Demonstrate free radical scavenging properties and anti-inflammatory activity *in vitro*	M(US & EU)	[8,82–85,124]
Promogran Prisma™ Matrix	Freeze-dried matrix composed of 55% bovine type I collagen with 44% ORC and 1% silver-ORC	Same benefits as Promogran™ Matrix Silver provides both antimicrobial and anti-inflammatory properties.	M(US & EU)	[8,82–85,124]
ColActive® PLUS	Porcine collagen, sodium alginate, CMC, EDTA and silver chloride	EDTA and collagen target and deactivate elevated MMP Activity	M(US & EU)	[125–127]
Suprasorb® X + PHMB	Biocellulose dressing made up of small-pored HydroBalance fibres that are produced using Acetobacter xylinium	Exudate control	M(US & EU)	[128–132]
BIOSTEP™ Collagen Matrix	Porcine gelatin and type I collagen matrix, EDTA, CMC and alginate	EDTA and collagen target and deactivate elevated MMPs activity	M(US)	[74,75]
Cutimed® Epiona	Fenestrated substrate made of 90% native bovine-derived collagen and 10% alginate	MMPs sequestered by the substrate due to collagen-binding properties.	M(US & EU)	[76,77]
Grafix®	Cryopreserved placental membrane, comprised of native viable cells, GFs and ECM	Retention of epithelial cells, fibroblasts and mesenchymal stem cells ↓ TNF-α and IL-1α ↑ IL-10	M(US)	[133–136]
Synthetic Dressings				
UrgoStart®	Soft-adherent foam dressing with TLC and NOSF	Neutralisation of excess proteases	M(EU)	[95–101]
PVA Sponge + MCG	Polyvinyl alcohol sponge impregnated with modified collagen gel	Promote shift of macrophage phenotype from ‘M1’ to ‘M2’ ↑ IL-10, IL-4 and VEGF	R	[31]
Drawtex®	Hydroconductive dressing obtained using LevaFibre™ technology, made of two absorbent, cross-action structures of viscose (63.2%) and polyester (26.8%)	Locks exudate and components away from the wound through capillary action ↓ MMPs levels	M(US & EU)	[29,30,137]
Biatain® Ag	Polyurethane foam with semi-permeable, bacteria- and top waterproof film	Exudate control Minimise risk of maceration and leakage	M(EU)	[138–141]
Dermagraft®	Polyglactin mesh with neonatal foreskin fibroblasts	Stimulate granulation tissue formation Stimulate secretion of cytokines and growth factors	M(US)	[142–146]

Legend: ↑: up-regulate; ↓: down-regulate, (US): approved to market in the United States; (EU): approved to market in the European Union; (US & EU): approved to market in both US and EU.

#### Skin substitutes

Skin substitutes are bioengineered dressings made of natural or synthetic polymers set to mimic the physiological geometry and function of native skin [47]. Skin substitutes for recalcitrant wounds are usually either acellular or cellular dermal components or dermo-epidermal components obtained through chemical synthesis of biological components or decellularisation of native ECM [48].

Integra® Dermal Regeneration Template (Integra Life Sciences Corporation, Plainsboro, New Jersey, US) was the first dermal skin substitute product approved by the US Food and Drug Administration (FDA). It consists of a porous matrix of cross-linked bovine tendon collagen type I, shark chondroitin-6-sulfate GAG and covered by a semi-permeable silicone membrane [47,49–53]. Chondroitin-6-sulfate GAG has been shown to have anti-inflammatory effect on macrophages; however, when the effects of Integra® on macrophage phenotype was analysed, a temporal down-regulation of ‘M2a’ macrophages (ECM deposition macrophages) was observed [54,55]. This is assumed to be due to the presence of glutaraldehyde as cross-linking agent in Integra® [54]. Nonetheless, clinical data show that Integra® induces deposition of collagen, histologically indistinguishable from native dermal collagen, achieving good quality tissue with no hypertrophic or keloid scar formation [54,56].

Decellularised xenografts have shown promising results in wound healing management, as they retain the native structure of the ECM, which comprises not only collagen but also structural, adhesion and signalling molecules (e.g. laminin, GAGs, elastin, fibronectin) [57,58]. Endoform® Antimicrobial Dermal Template (Aroa Biosurgery Ltd., Auckland, NZ) presents a matrix of ovine forestomach (OFM) with 0.3% w/w silver chloride [59,60]. Its main component, OFM, has been shown to promote wound healing in recalcitrant wounds (wound closure achieved within 4–24 weeks) with no reports of adverse reactions when used as Endoform® Dermal Template [61–63]. OFM/silver has been demonstrated to be effective at inhibiting a broad spectrum of MMPs and exhibits low cytotoxicity *in vitro* [32,57,64]. This large MMP-spectrum is assumed to be due to the retained native ECM structure [32,57,64]. A preliminary *in vivo* study showed positive results on wounds characterised by different aetiologies [65].Puracol® Ultra ECM (Puracol® Ultra ECM, Medline Industries Inc., Northfield, IL) is a decellularised porcine mesothelium matrix that has also been shown to have a high retention of growth factors (FGF-basic, VEGF, and TGF-β1) after the decellularisation process, high angiogenetic potential *in vitro* and similar MMPs-inhibition abilities to OFM/silver [58]*.* Although more research is needed on skin substitutes’ full effects in curbing inflammation, they represent an attractive, albeit expensive, way of modulating inflammation and promoting healing [66].

#### Collagen dressings

Collagen plays many roles in wound management, including chemotaxis of fibroblasts, wound contraction, induction of growth factors and cytokines, activation and inhibition of MMPs [67,68]. Together with its biocompatibility, biodegradable, and non-toxic attributes, these advantages make collagen an attractive candidate material for treating recalcitrant wounds. Numerous clinical studies highlight the benefit of collagen-based dressings for treating chronic wounds, showing faster healing rates or shorter healing times, inactivation of proteases and maintenance of moist wound environment [69–72].

There are many commercially available collagen-based wound dressings, all with different composition but similar claims. They are made of collagen or a combination of collagen and other ECM components, such as elastin, hyaluronic acid and chitosan or in conjunction with other biologically derived materials like alginate and cellulose [73]. BIOSTEP™ Collagen Matrix dressing (Smith & Nephew, London, U.K.) is a matrix presenting both type I and denatured (gelatin) porcine collagen with the addition of EDTA, CMC and alginate [74]. The collagen acts as a sacrificial layer for excess MMPs, while EDTA binds and permanently inactivate them [74,75]. The presence of both collagen and gelatin is claimed to attract both collagenase (MMP-1) and gelatinase (MMP-2, MMP-9) [74]. Cutimed® Epiona (Essity Medical Solutions, Stockholm, Sweden) is a fenestrated substrate made of 90% native bovine-derived collagen and 10% alginate [76,77]. The dressing structure is claimed to be nearly identical to human dermis, which allows for MMP binding and reduction in inflammation [76,77]. Despite the common use of collagen-based wound dressings in wound care, more research is needed to understand their mechanisms of action. Many dressings lack satisfactory evidence-based data due to poorly-designed studies that, in many cases, are industry-funded or present traditional dressing (i.e. saline moistened gauze) as control [69,70].

#### Cellulose

Cellulose is a naturally occurring polymer used in biomedical applications for its biocompatibility, biodegradability, low-toxicity and good absorption properties [78,79]. There are different cellulose sources and derivatives. An example is given by oxidised regenerated cellulose (ORC), a chemically modified form of cellulose with haemostatic abilities [80,81]. The Promogran™ Matrix family (3M, Saint Paul, MN, US) of wound dressings are collagen-based dressings containing ORC (3M™ Promogran™ Protease Modulating Matrix) or ORC and silver-ORC (3M™ Promogran Prisma™ Wound Balancing Matrix). Promogran™ Matrix is composed of a freeze-dried matrix made of 55% type I bovine collagen and 45% oxidised regenerated cellulose (ORC), whereas Promogran Prisma™ Matrix replaces 1% of ORC with silver-ORC to provide the dressing with antimicrobial properties. The combination of collagen and ORC presents a series of anti-inflammatory benefits. Firstly, ORC has been shown to passively affect protease levels through its negative charge that attracts positively charged metal ions which are essential for the activation of MMPs and thereby reduces MMPs activity [82]. ORC has the additional benefit of reducing elastase activity, an enzyme that breaks down elastin fibres within the ECM and contributes to non-healing wounds’ chronicity. Studies have shown that collagen/ORC dressings reduce elastase activity and inhibit MMP-2 and MMP-9 activity in chronic wound exudate, resulting in increased healing rates [83,84]. Additionally, collagen/ORC binds platelet-derived growth factor (PDGF) and shields it from degradation within the wound and γ-irradiation when loaded into the dressing during the manufacturing process, opening a series of opportunities to locally deliver exogenous growth factors and protection of endogenous growth factors within the wound [82,83]. These benefits have also been analysed and recognised by many clinical reviews [8,85].

#### Functionalised collagen dressings

Numerous studies showed functionalisation of collagen-based dressings incorporating growth factor or peptides to provide a more instructive wound microenvironment to aid cellular behaviour and tissue regeneration [86]. An example is given by a chitosan-collagen hydrogel which incorporates an angiopoietin-1 (Ang1) mimetic peptide, QHREDGS (glutamine-histidine-arginine-glutamic acid-aspartic acid-glycine-serine) [33,86–88]. Ang1 has been widely recognised to positively participate in several cellular processes such as vascular protection, wound healing and inflammation, providing skin cells protection from oxidative stress [87]. This product is in development as an injectable gel and a pre-gelled patch and presents the QHREDGS peptide conjugated to the dressing's chitosan component to avoid systemic circulation of the peptide and to promote a more localised activity [88]. The QHREDGS-functionalised hydrogel has been shown to improve *in vitro* keratinocytes resistance to oxidative stress caused by elevated ROS levels, which is common in diabetic chronic wounds [87]. In addition, when co-cultured with macrophages, the Q-peptide hydrogel induces a shift in macrophages polarisation, resulting in the expression of both pro-inflammatory and anti-inflammatory cytokines; it, therefore, offers the potential to modulate the stalled inflammatory process in chronic wounds [33].

Growth factors have also shown promising results when included in a scaffold as topical therapies [34,89,90]. Long et al. [34] produced a collagen scaffold co-modified with VEGF and SDF-1α. These were loaded onto the scaffold after separately being fused with a collagen-binding domain (CBD); this allows for a more controlled release of the growth factors once implanted [34]. This co-modified CBD-VEGF-SDF-1α collagen scaffold showed reduced infiltration of ‘M1’ macrophages together with reduced expression of IL-1β and TNF-α, two pro-inflammatory cytokines found at high levels in chronic wounds [34]. Furthermore, the synergic action of VEGF and SDF-1α promotes blood vessel formation, which is thought to reduce hypoxia at the wound site [34].

#### Silver

Ionic silver (Ag+) has been shown to provide anti-inflammatory properties in addition to its antimicrobial activity, although the mechanism of action is not well understood [15,91]. A silver nanoparticle loaded collagen/chitosan scaffold (NAg-CSS) claimed to modulate fibroblast migration and macrophage activation to promote healing has been devised by You and colleagues [35]. Compared with non-loaded collagen/chitosan scaffold, NAg-CSS significantly decreased the expression of CD68 (a macrophage marker), further proven by the inhibition of pro-inflammatory cytokines IL-6, TNF-α and TGF-β while up-regulating the anti-inflammatory cytokines, IL-10 and IFN-γ [35]. Even though silver nanoparticles’ exact anti-inflammatory mechanism is not yet fully understood, the different NAg-CSS components’ combinations could offer a synergic effect. Chitosan has been shown to stabilise collagen scaffolds’ mechanical properties while also providing practical benefits such as antioxidant and antimicrobial properties [92].

### Synthetic wound dressings to regulate inflammation

Synthetic wound dressings are considered not as competitive as bio-derived as they do not mimic the native EMC in a similar manner to bio-derived dressings [19]. The most common types of polymers used in wound dressings include polyurethane, polyester, poly(glycolic acid), poly-l-lactide and poly(lactic-co-glycolic acid) [93,94]. Compared with bio-derived dressings, synthetic dressings present advantages in terms of reproducibility and tailoring as their synthesis is more easily controlled. However, they tend to be more inert, not provide a physiological microenvironment to promote healing, and consideration needs to be given to degradation components left in the host tissue [37,41].

Refer to Table 2 for a summary of dressings currently available or in the research stage.

#### Technology lipido colloid (TLC)

The synthetic dressing UrgoStart® (Urgo Medical, Chenôve, France) presents a polyester mesh saturated with a sucrose octasulfate potassium salt (Nano Oligo Saccharide Factor, NOSF) embedded lipido-colloid matrix (Technology Lipido-Colloid, TLC). NOSF and TLC composition are protected under the manufacturer's patent. UrgoStart (TLC-NOSF) turns into a colloidal solution, allowing for conformability to the wound bed [95]. Oligosaccharides (NOSF) have been shown to reduce MMPs level and restore growth factors biological functions, while the TLC matrix creates a moist wound environment [96]. Furthermore, *in vitro* data report decreased levels of gelatinases and an initial decrease of collagenases (MMP-1 and MMP-8) [97]. Multiple clinical studies report its effectiveness in DUs, VUs and PUs; however, there is a lack of data regarding its mechanism of action [96,98–101].

#### Polyvinyl alcohol (PVA) sponge

An exciting example of a collagen-functionalised synthetic dressing is presented by Das and co-workers [31]. The authors saturated a polyvinyl alcohol (PVA) sponge with a modified collagen gel (MCG), implanted it subcutaneously in a mouse model and assessed the effects on inflammation after 3 and 7 days [31]. They found MCG increased macrophage recruitment *in situ*, decreased pro-inflammatory ‘M1’ phenotype and promoted an ‘M2’ phenotype [31]. This was further proved by the increased levels of anti-inflammatory IL-10 and IL-4 and pro-angiogenic VEGF production [31]. Furthermore, they showed MCG induces IL-10 production via the miR-21-JNK pathway; however, the lack of testing in a wound model calls for further research [31].

## Summary

- Wound treatment represents one of the most expensive burdens on healthcare systems worldwide.
- Many chronic wounds become stuck in the inflammatory phase, which prevents healing from progressing.
- A vast market of wound dressings presents different composition but similar claims, making it difficult for healthcare practitioners to decide the appropriate treatment.
- Collagen is one of the most used materials in wound dressings since it helps mimic the native wound microenvironment.
- Additional research is needed to fully understand the mechanisms of action of collagen-based wound dressings to curb inflammation, and a better clinical study design needs to be implemented so that the results obtained are truly valuable.

## Abbreviations

Ang1: angiopoietin-1
CBD: collagen binding domain
CMC: carboxymethylcellulose
DAMPs: danger-associated molecular patterns
DFUs: diabetic foot ulcers
ECM: extracellular matrix
EDTA: ethylenediaminetetraacetic acid
FDA: US Food and Drug Administration
GAGs: glycosaminoglycans
IFN-γ: interferon-γ
IL-x: interleukin-x (e.g. IL-6, IL-10, IL-4, etc.)
LUs: leg ulcers
MCG: modified collagen gel
MMPs: matrix metalloproteinases
NHS: UK National Health Services
OFM: ovine forestomach matrix
ORC: oxidised regenerated cellulose
PAMPs: pathogen-associated molecular patterns
PDGF: platelet-derived growth factor
PHMB: polyhexamethylene biguanide
Pus: pressure ulcers
QHREDGS: glutamine–histidine–arginine–glutamic acid–aspartic acid–glycine–serine
ROS: reactive oxygen species
SDF-1α: stromal cell-derived factor-1α
TGF-β: tissue growth factor-β
TIMPs: tissue inhibitors of metalloproteinases
TNF-α: tumour necrosis factor-α
VEGF: vascular endothelial growth factor
